# Supplementary figures and images for: Integrated multi-omic analyses provide insight into colon adenoma susceptibility modulation by the gut microbiota
Source: mSystems. 2023 Jul 17;8(4):e00151-23. doi: 10.1128/msystems.00151-23 (PMC10469915; doi:10.1128/msystems.00151-23)

a

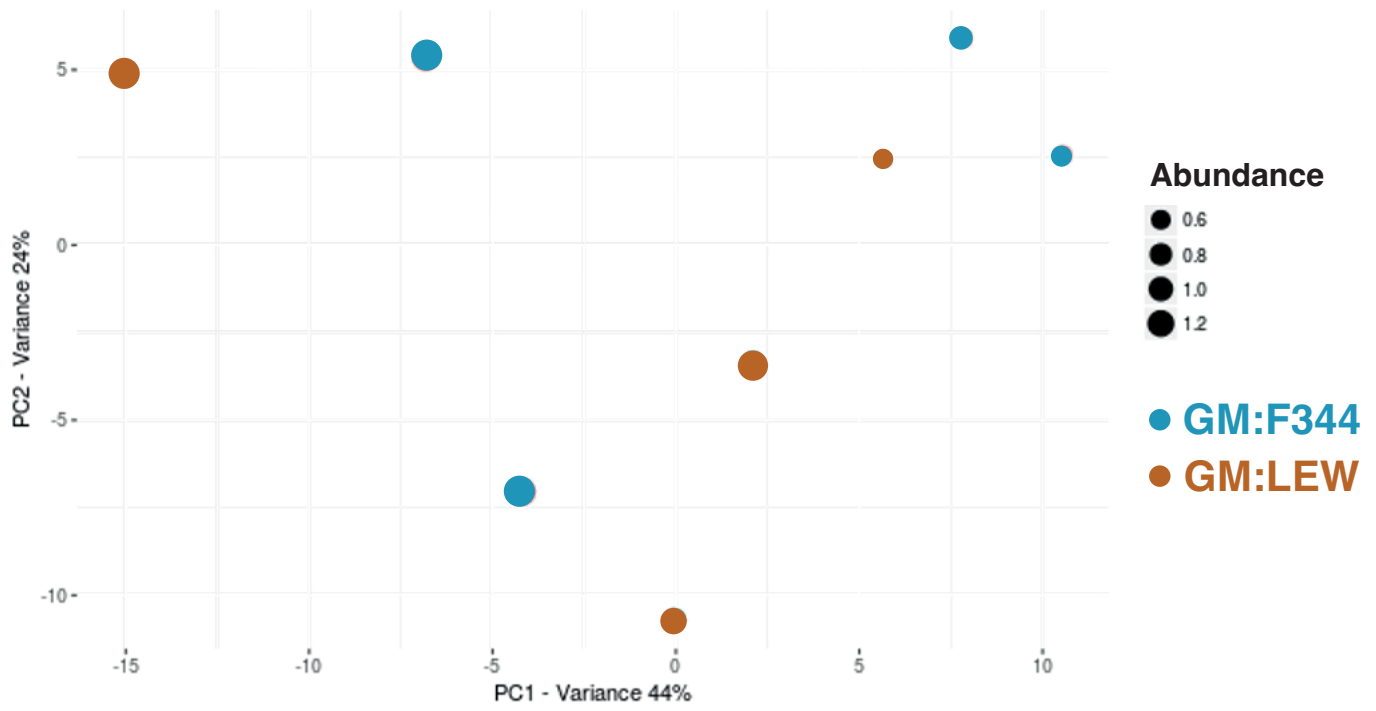

b

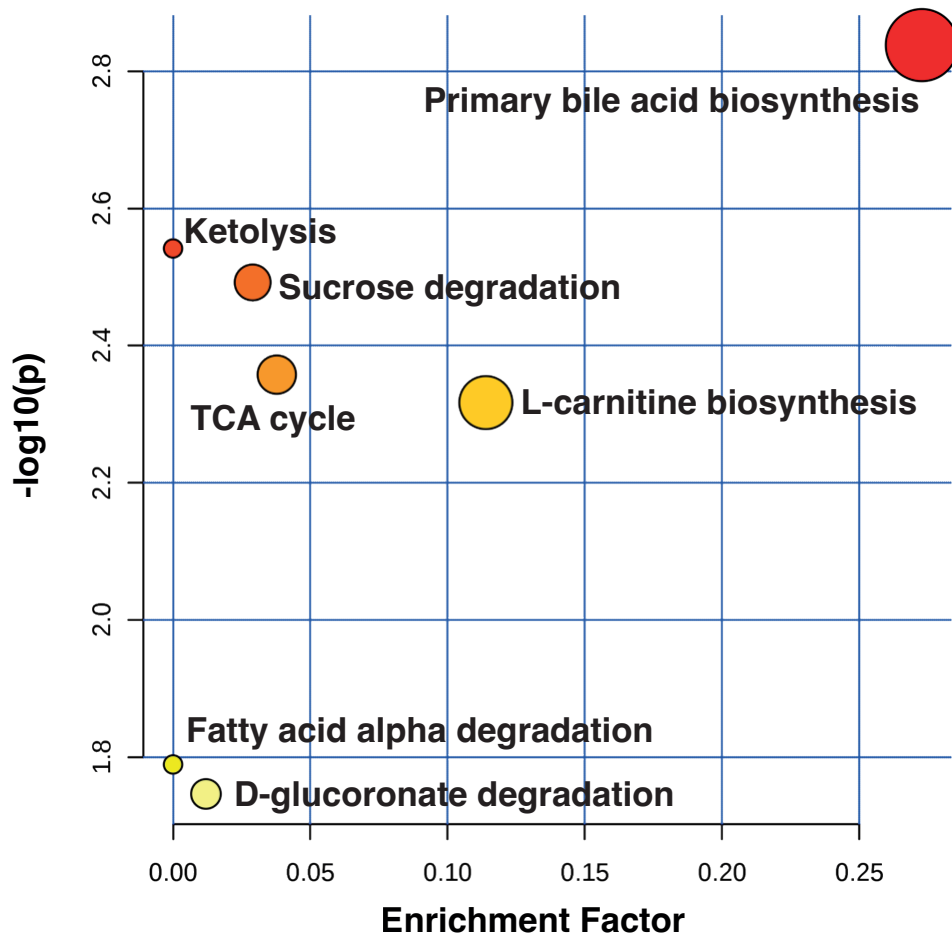

Supplement: Fig. S1 — Serum metabolomics profiles and pathway analyses in Pirc and WT rats. [file msystems.00151-23-s0001.pdf]

a

## GM:F344

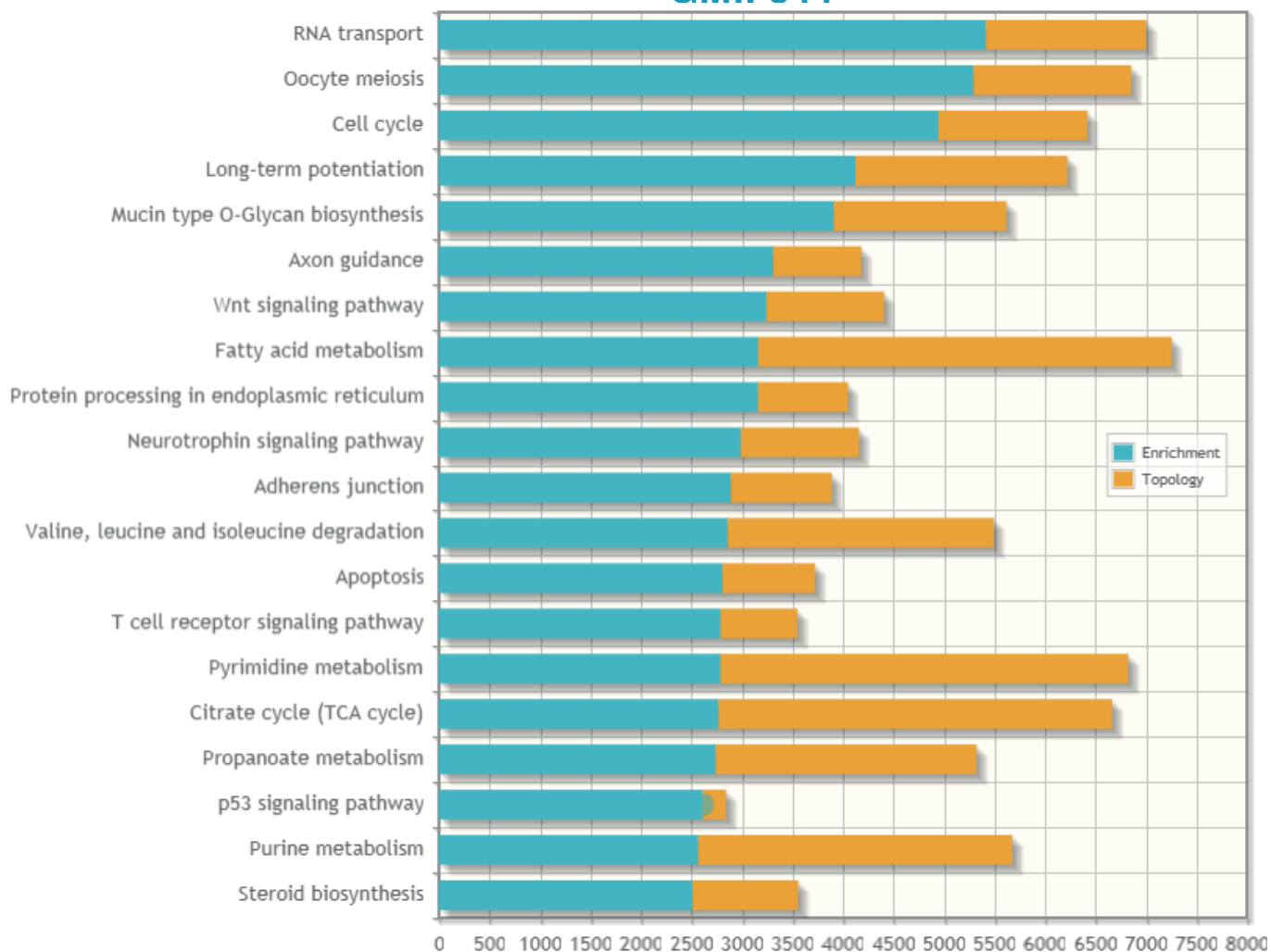

b

## GM:LEW

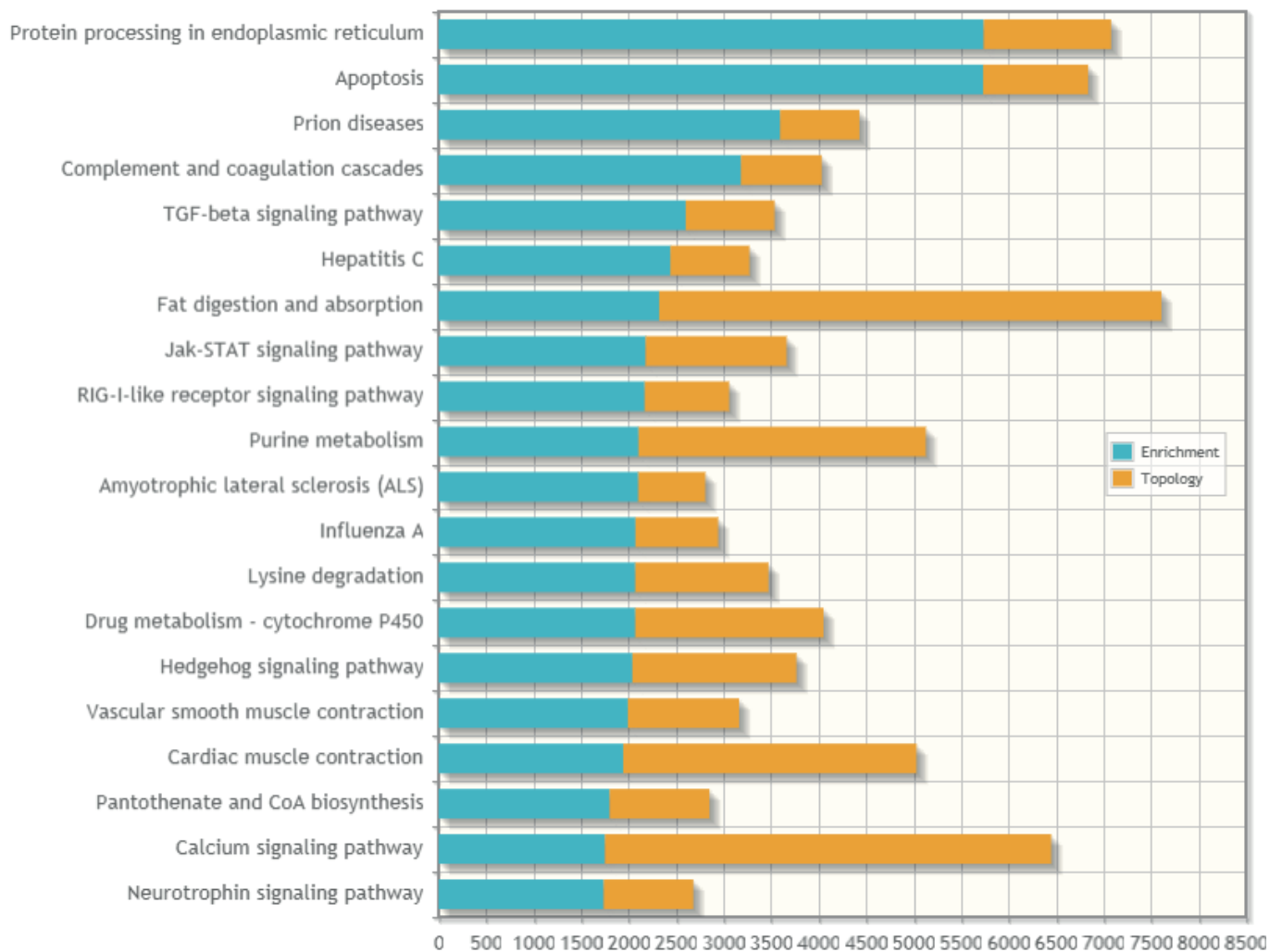

Supplement: Fig. S2 — Differentially expressed genes (DEGs) and pathways altered due to GM in the normal epithelium and tumor tissues. [file msystems.00151-23-s0002.pdf]

a

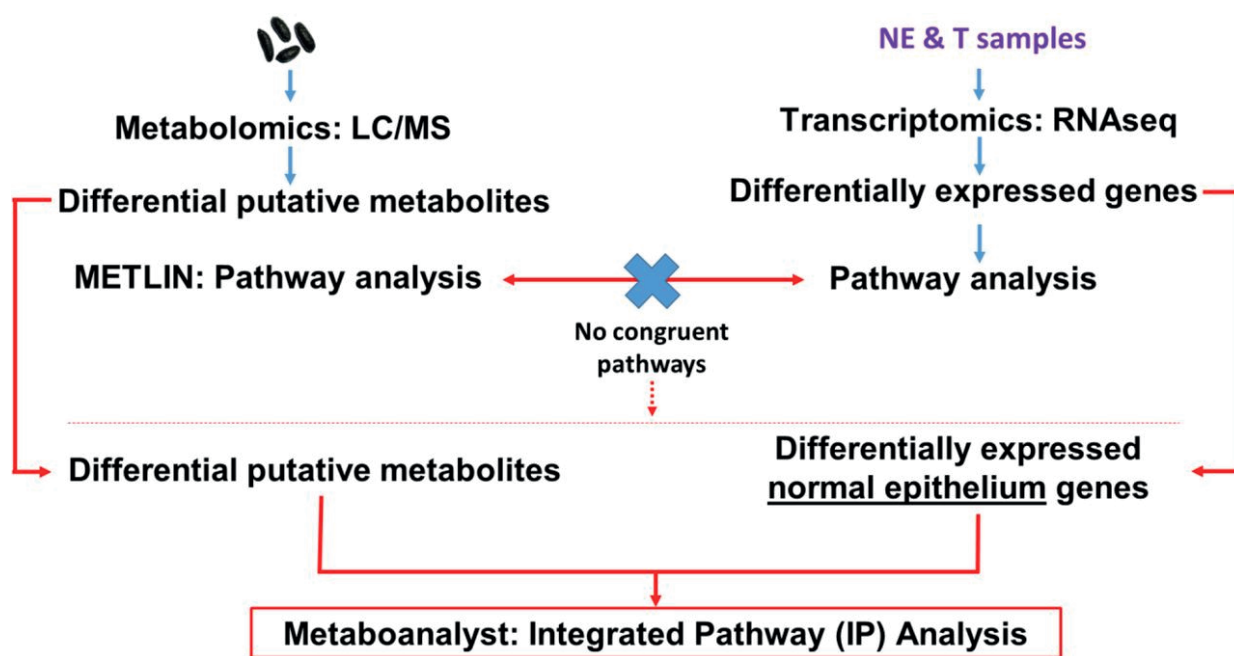

b

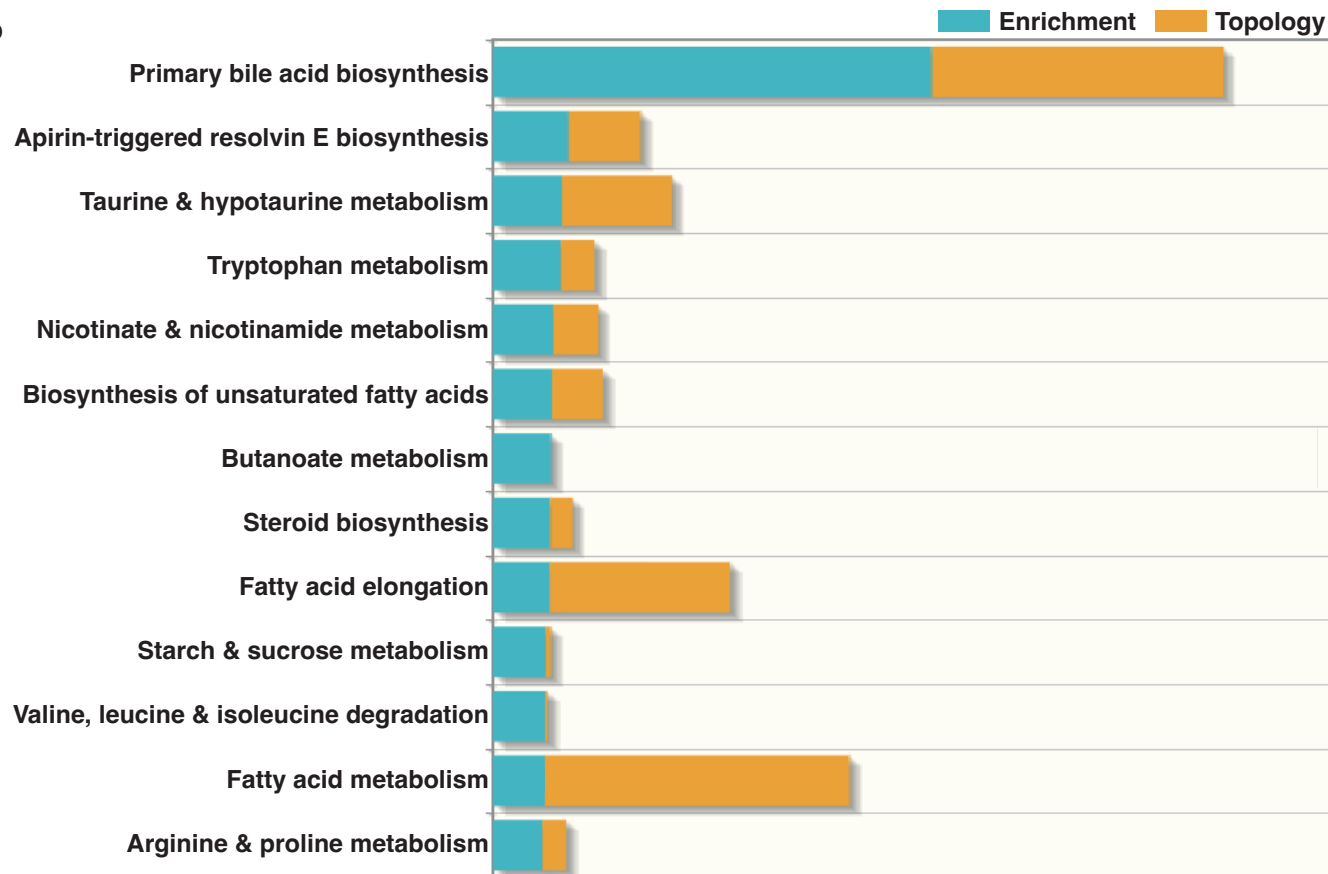

Supplement: Fig. S3 — Multi-omic integrated analysis. [file msystems.00151-23-s0003.pdf]

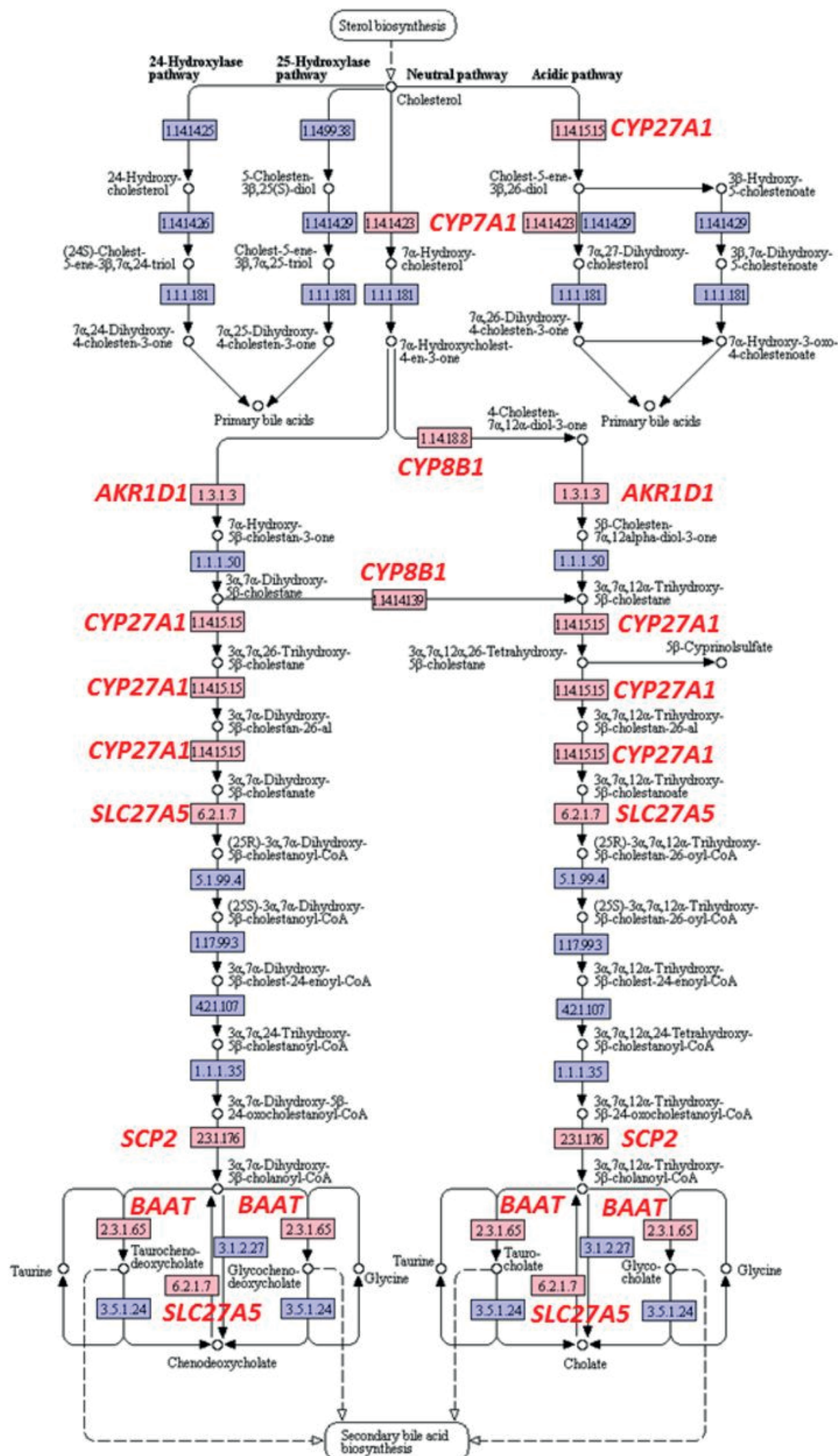

Supplement: Fig. S4 — Bile acid biosynthesis pathway. [file msystems.00151-23-s0004.pdf]

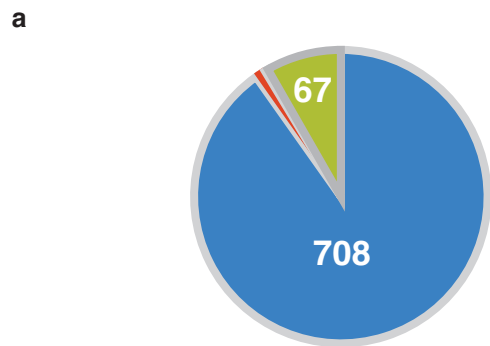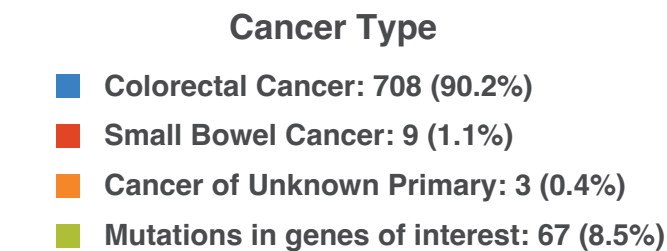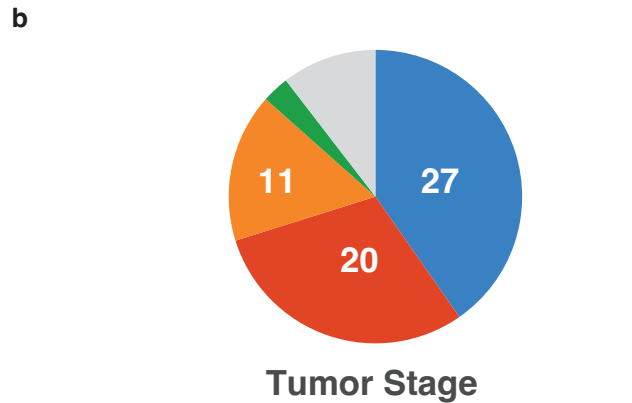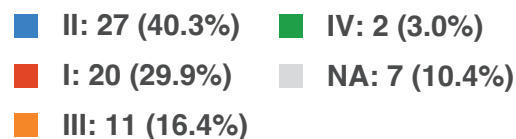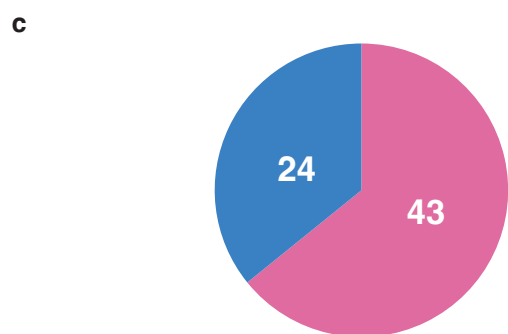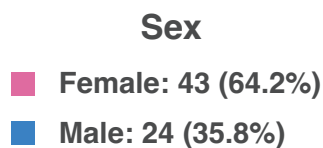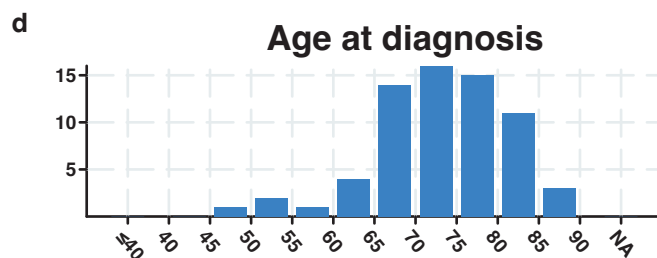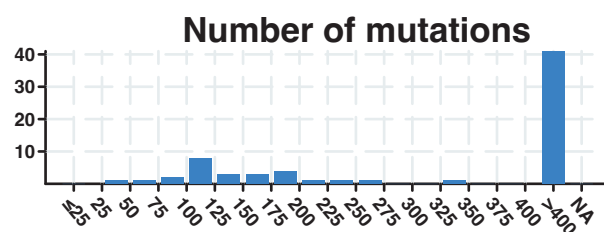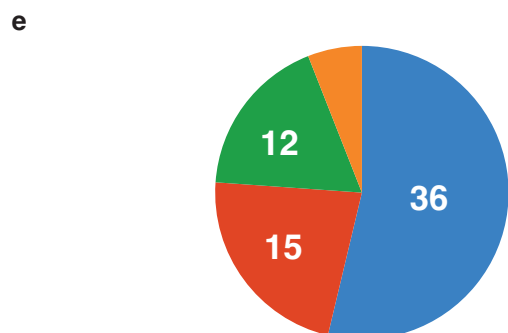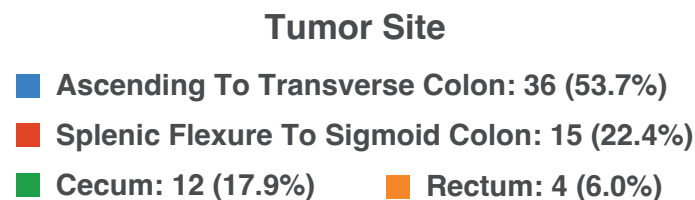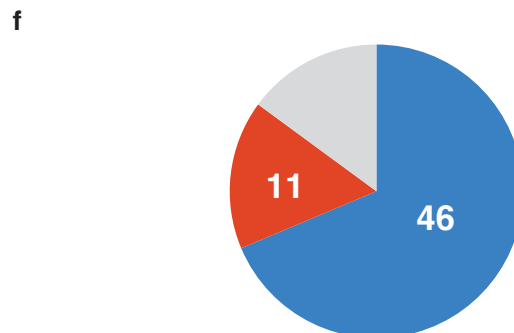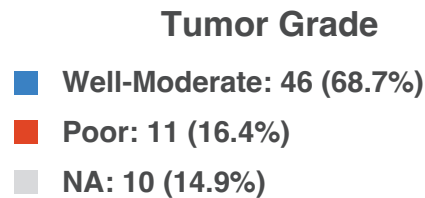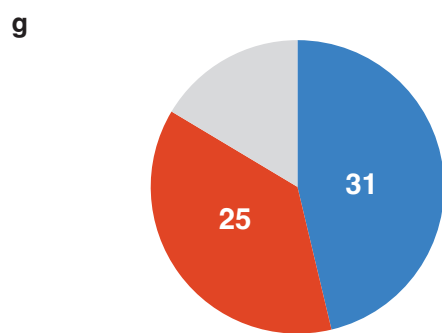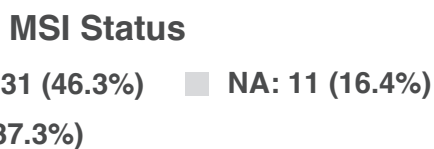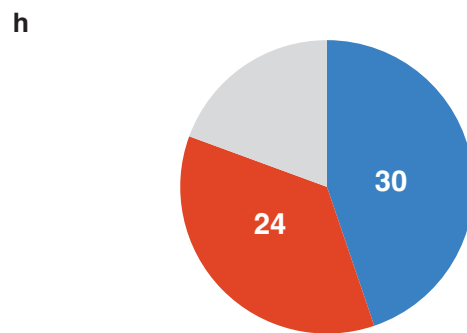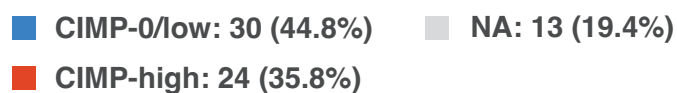

Supplement: Fig. S5 — Genes of interest identified in The Cancer Genome Atlas (TCGA). [file msystems.00151-23-s0005.pdf]
