# Supplementary material for: Integrated multi-omic analyses provide insight into colon adenoma susceptibility modulation by the gut microbiota
Source: mSystems. 2023 Jul 17;8(4):e00151-23. doi: 10.1128/msystems.00151-23 (PMC10469915; doi:10.1128/msystems.00151-23)
Supplement: Supplemental Information — Supplemental figure and table legends. [file msystems.00151-23-s0006.pdf]

Supplementary information

Integrated multi-omic analyses provide insight into colon adenoma susceptibility modulation by the gut microbiota

Susheel Bhanu Busi[1,2], Zhentian Lei[3,4], Lloyd W. Sumner[3,4], and James M. Amos-Landgraf[1,5,6]

Supplementary figure legends

Supplementary Figure 1. Serum metabolomics profiles and pathway analyses in Pirc and WT rats

Serum samples collected from Pirc and WT rats at 1-month of age used for LC-MS analysis indicated differential metabolomics profiles (a) including the regulation of bile acid biosynthesis, L-carnitine biosynthesis and fatty acid alpha-oxidation as potential pathways (b), contributing to phenotypic differences.

Supplementary Figure 2. Differentially expressed genes (DEGs) and pathways altered due to GM in the normal epithelium and tumor tissues

Pathway analyses based on the expression of upregulated genes in the normal tissues of the GM:F344 (a) and GM:LEW (b) groups was used to identify potential pathways and mechanisms contributing to the low and high tumor susceptibility. Enriched pathways are indicated in blue, while the topology, i.e. the importance of the pathway to the overall phenotype observed is shown in yellow. MetaboAnalyst Integrated pathways (IP) analysis incorporating the differentially expressed genes and the putative metabolites, significantly different between GM:F344 and GM:LEW, was performed.

Supplementary Figure 3: Multi-omic integrated Analysis

(a) Metabolomics and gene expression results were used to generate host pathways associated with changes in the GM. The two analyses did not share pathways of interest. To address the incongruence, the differential putative fecal metabolites and

normal epithelium genes were used to generate an integrated pathway analysis. (b) Integrated pathway analyses depicts pathways enriched and their topology, contributing to the variability in tumor phenotype observed as an effect of the genes and metabolites.

Supplementary Figure 4: Bile acid biosynthesis pathway

Genes identified via metabolomics and RNAseq analysis, contributing to the bile acid pathway analyses are identified by highlighting corresponding locations in the KEGG pathway. The pathway was built using the KEGG pathway mapper tool from www.genome.jp/KEGG. The pathway image is reproduced with permission from KEGG.

Supplementary Figure 5: Genes of interest identified in The Cancer Genome Atlas (TCGA)

Genes identified via metabolomics and RNAseq analysis, contributing to the bile acid pathway along with *ALOX5* and *PTGS2* were used to query the TCGA dataset. (a) Pie chart depicting the total number of Colonic Adenocarcinoma samples in TCGA and the samples (8.5%, i.e n=67/785) carrying mutations in genes identified in the integrated analyses. The tumor stage (b), sex (c), age at diagnosis (d - top) and mutations (d - bottom) are depicted. (e) The tumor sites, (f) grade, microsatellite instability (g; MSI), and the CpG Island Methylator Phenotype (h; CIMP) are depicted for the 67 samples are indicated.

Supplementary table legends

Supplementary Table 1: Summary of data processing results

The raw peaks obtained via XCMS for each individual samples analyzed through LC-MS is shown with an average peak abundance in the samples being 497. The number of missing or zero peaks for each sample along with the number of peaks processed for analysis based on the cutoff established in the Methods sections are listed. The raw data for the metabolomics analyses is hosted through the Metabolomics

Workbench on the NIH Metaboloics Data Repository under the DataTrack ID #1539 for public access.

Supplementary Table 2: Differentially expressed genes in the normal epithelium and tumor tissues of GM:F344 and GM:LEW

Genes differentially expressed between the groups in the normal epithelium and the tumors.
